# *Cryptosporidium* and *Giardia* in Surface Water: A Case Study from Michigan, USA to Inform Management of Rural Water Systems

**DOI:** 10.3390/ijerph111010480

**Published:** 2014-10-13

**Authors:** Erin A. Dreelin, Rebecca L. Ives, Stephanie Molloy, Joan B. Rose

**Affiliations:** 1 Department of Fisheries and Wildlife and Center for Water Sciences, Michigan State University, 301 Manly Miles Building, 1405 S. Harrison Road, East Lansing, MI 48823, USA; 2 Department of Fisheries and Wildlife, Michigan State University, 480 Wilson Road, East Lansing, MI 48823, USA; E-Mails: ivesrebe@msu.edu (R.L.I.); rosejo@msu.edu (J.B.R.); 3 Environmental Services Department, City of San Jose, 200 E. Santa Clara St. 10th Floor, San Jose, CA 95113, USA; E-Mail: stephanie.molloy@sanjoseca.gov

**Keywords:** *Cryptosporidium*, *Giardia*, genotyping, land use, rural water supply, water management

## Abstract

*Cryptosporidium* and *Giardia* pose a threat to human health in rural environments where water supplies are commonly untreated and susceptible to contamination from agricultural animal waste/manure, animal wastewater, septic tank effluents and septage. Our goals for this paper are to: (1) explore the prevalence of these protozoan parasites, where they are found, in what quantities, and which genotypes are present; (2) examine relationships between disease and land use comparing human health risks between rural and urban environments; and (3) synthesize available information to gain a better understanding of risk and risk management for rural water supplies. Our results indicate that *Cryptosporidium* and *Giardia* were more prevalent in rural *versus* urban environments based on the number of positive samples. Genotyping showed that both the human and animal types of the parasites are found in rural and urban environments. Rural areas had a higher incidence of disease compared to urban areas based on the total number of disease cases. Cryptosporidiosis and giardiasis were both positively correlated (*p* < 0.001) with urban area, population size, and population density. Finally, a comprehensive strategy that creates knowledge pathways for data sharing among multiple levels of management may improve decision-making for protecting rural water supplies.

## 1. Introduction

*Cryptosporidium* and *Giardia* have caused multiple high-profile outbreaks around the globe and are a major concern for water safety because they are resistant to chemical disinfection and are highly infectious. Of the 199 waterborne disease outbreaks reported globally between 2004 and 2010 that were caused by parasitic protozoa, *Cryptosporidium* and *Giardia* were the most common etiological agents [1]. *Cryptosporidium* is responsible for approximately 20% of diarrheal cases in children in developing countries and up to 9% of cases in developed countries, whereas *Giardia* is responsible for 20–30% in developing countries and 2–7% of cases in developed countries [2]. The pathogens are transmitted by the fecal-oral route via human-human contact, human-animal contact, contaminated food, or contaminated water. For this paper, we focus on the contamination of water supplies by the parasites. Because of the difficulty with treating source water to eliminate these protozoan parasites in drinking water, it is important to understand the sources, fate and transport in the environment [3].

*Cryptosporidium* and *Giardia* may be introduced into waterbodies by point or nonpoint (diffuse) pollution sources. Human sources of *Cryptosporidium* and *Giardia* contamination may include untreated or improperly treated sewage, discharges of untreated sewage via sanitary sewer overflows or combined sewer overflows (CSOs), land application of biosolids and septage, and leaking sewer or septic tank and drainage systems. Animal sources include runoff from domestic livestock operations or fields fertilized using animal manure, defecation by pets in the environment, and defecation by wildlife. Wildlife can contribute to *Cryptosporidium* contamination in water; however, wildlife sources may not be a major public health concern due to host-specificity [4]. In the case of giardiasis, the zoonotic transmission route of exposure does not appear to be as significant as compared to *Cryptosporidium* [5]. Poor sanitation and hygiene are the key exposure risks for giardiasis, particularly in undeveloped countries [5]. In general, fecal contamination of waterways from human sources and livestock may be of greater human health concern than contamination by wildlife [4,6].

Rural and urban areas differ in the occurrence, distribution, and concentration of potential sources of fecal contamination. Rural areas are typically characterized by lower human population densities, higher domesticated livestock densities, and decentralized drinking water and sanitation systems. In contrast, urban areas are typically characterized by high human population densities, a high percentage of developed or artificial land cover, and more highly engineered drinking water and sanitation infrastructure. Thus, populations in rural and urban areas may face different health risks from protozoan parasites. Studies across the globe, such as those conducted in Switzerland [7], Spain [8,9], Hungary [10], China [11], and Paris [12], have implicated agricultural activities and livestock as major sources of protozoan parasites found in source waters. Few studies have examined how land use and population characteristics are related to the presence of *Cryptosporidium* and *Giardia* or the incidence of cryptosporidiosis and giardiasis. In Scotland, increased rates of *C. parvum* infection were found in areas with lower human density, a higher ratio of farms to humans, and a higher ratio of private water supplies to the human population, indicating an association of *C. parvum* infection with rural areas [13]. In England and Wales, rates of cryptosporidiosis were higher in rural areas (defined by housing density), areas with more agricultural manure application, and areas with poorly treated water supplies [14]. However, only weak relationships were observed between giardiasis rates and cattle density and between giardiasis rates and land application of animal manure in a Canadian study [15].

In this paper, we use a case study from Michigan, USA to examine protozoan parasites in water systems across an urban-rural gradient. Michigan provides a useful case study because the state encompasses a diversity of land uses, types of source water, and types of water systems. For the case study, we use a combination of observational data and literature to explore the risks from *Cryptosporidium* and *Giardia* and implications for management based on two main lines of evidence: the prevalence of the parasites and the incidence of disease. Our goals are to explore three main questions: (1) How prevalent are *Cryptosporidium* and *Giardia* in rural *versus* urban environments? (2) What relationships have been observed between disease, land use, and other environmental factors? and (3) What are the implications for protecting rural waters? We hypothesize that *Cryptosporidium* is typically more prevalent and poses more risk in rural areas whereas *Giardia* is a bigger concern in urban areas.

## 2. Experimental Section

### 2.1. Case Study Background: Michigan, USA

The state of Michigan lies within the Laurentian Great Lakes watershed and is surrounded by Lakes Superior, Michigan, Huron and Erie. Michigan is home to approximately 9.8 million people as of 2013 [16]. Based on recent land cover estimates, approximately 5% of the state is urban, 26% agriculture, and 38% upland forest with the remainder in lowland forest, wetland, open land, and non-vegetated land cover types [17]. The main urban areas in the state are the Detroit metro area and the cities of Grand Rapids, Flint, Lansing, and Ann Arbor; all of these population centers are located in the southern portion of the Lower Peninsula. The remainder of the state is rural, with approximately 19% of the population and 75% of the landmass classified as rural by the Michigan Department of Community Health [18].

Michigan has approximately 1425 community water systems and over 10,000 non-community water systems that are regulated under Safe Drinking Water Act programs by the Michigan Department of Environmental Quality (MDEQ) with oversight from the US Environmental Protection Agency (EPA) [19]. The state has the highest number of private wells in the US, with over 1 million households served [19]. The MDEQ works with local health departments to regulate well construction and safe decommission and requires that private drinking water wells be tested for indicator bacteria after installation and prior to use; however, additional water quality monitoring requirements vary by county and are determined by local health departments.

Groundwater is the most common source of drinking water in Michigan based on the number of systems, with approximately 97% of public water systems using groundwater as reported in the EPA SDWIS database [20]. However, most of the large urban areas in the state, including the cities of Detroit, Grand Rapids, Flint and Ann Arbor, obtain drinking water from surface water sources. As a result, approximately 65% of Michigan’s population is served by surface water. These patterns in land use and water systems result in rural populations that are typically served by private wells with little to no monitoring and treatment, whereas urban water systems are treated, monitored, and carefully regulated (Table 1).

**Table 1:** Comparison of Michigan rural and urban communities.

Characteristic	Rural	Urban
Source Water	Groundwater	Surface water
Common Water System Type	Private wells	Community systems
Dominant Waste Streams	Livestock	Human
Drinking Water Regulations	Minimal regulation once wells are constructed	Safe Drinking Water Act requirements enforced

**Figure 1:**
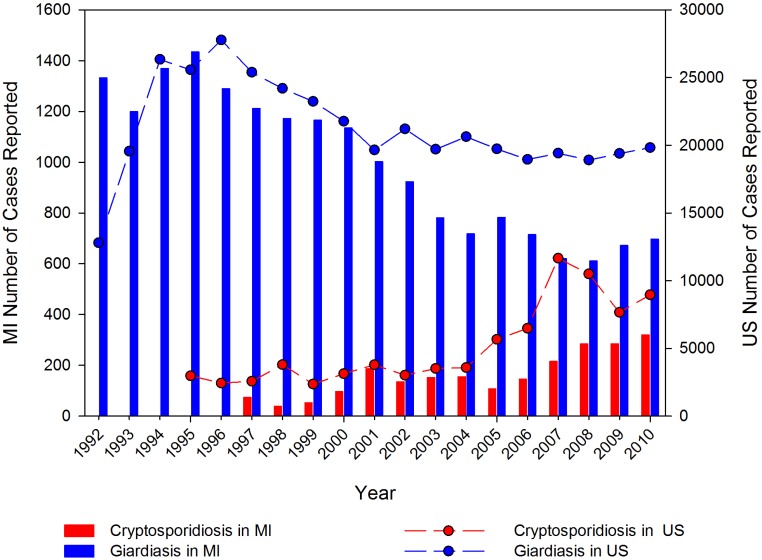
Annual number of cases of cryptosporidiosis (red) and giardiasis (blue) reported in the United States (lines) and Michigan (MI; bars) from 1992–2010. Cryptosporidiosis reporting for the US began in 1995 with 27 states reporting data; Michigan began reporting cryptosporidiosis in 1997. Note the difference in scales for Michigan (Y1) and US (Y2) cases. (Data sources: [22,23,24]).

Michigan began reporting cases of giardiasis and cryptosporidiosis in 1992 and 1997, respectively. Cases of giardiasis are more common than cryptosporidiosis, which is similar to national trends (Figure 1). Cryptosporidiosis is most commonly reported for the 0-9 age group; 25% of reported cases between 2003 and 2007 occurred in this group [21]. For giardiasis, approximately half of the cases reported are in people under 19, with 31% of the cases occurring in the 0-9 age group during the same time period [15]. As with the national data, the number of cases of giardiasis in Michigan peaked in the mid-1990s and has declined, whereas cryptosporidiosis cases have increased since the 1990s.

### 2.2. Prevalence in the Environment

**Figure 2:**
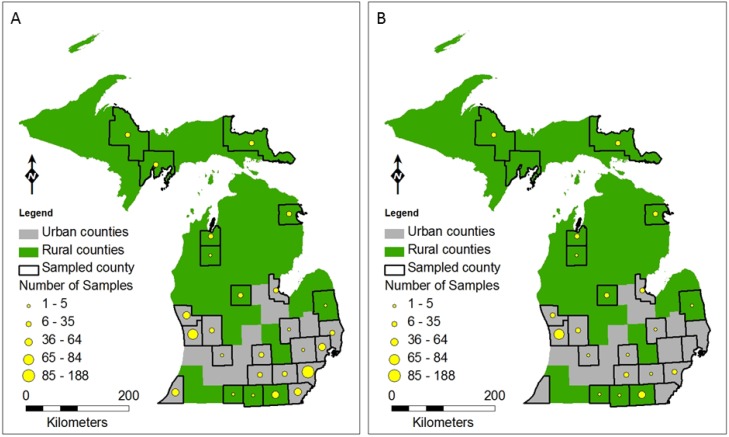
Map of the sampling sites for (**A**) *Cryptosporidium* and (**B**) *Giardia* in Michigan, USA. Green areas are classified as rural and gray areas are classified as urban by the Michigan Department of Community Health [18]. Black borders highlight counties that were sampled for either *Cryptosporidium* or *Giardia*. Circles within county boundaries indicate intensity of monitoring based on the number of surface and groundwater samples collected for each county.

We used several data sources to assess prevalence of *Cryptosporidium* and *Giardia* across Michigan. We compiled all data from multiple studies conducted in Michigan by our lab, the Michigan State University (MSU) Water Quality, Environmental, and Molecular Microbiology Laboratory. These data include samples from private wells and surface waters in both urban and rural areas of the state (Figure 2). In addition to these field data, we also downloaded results from the USEPA Long Term 2 Enhanced Surface Water Treatment Rule (LT2ESWTR or LT2) monitoring program [25] and extracted data for Michigan. The LT2 data consist of raw water samples collected at intakes of drinking water plants using surface water or groundwater directly under the influence of surface water which were analyzed for *Cryptosporidium* following USEPA Method 1622 [26] or 1623 [27]. *Giardia* data were included from LT2 samples analyzed by the MSU Water Quality, Environmental, and Molecular Microbiology Laboratory.

#### 2.2.1. Field Sampling Sites

**Figure 3:**
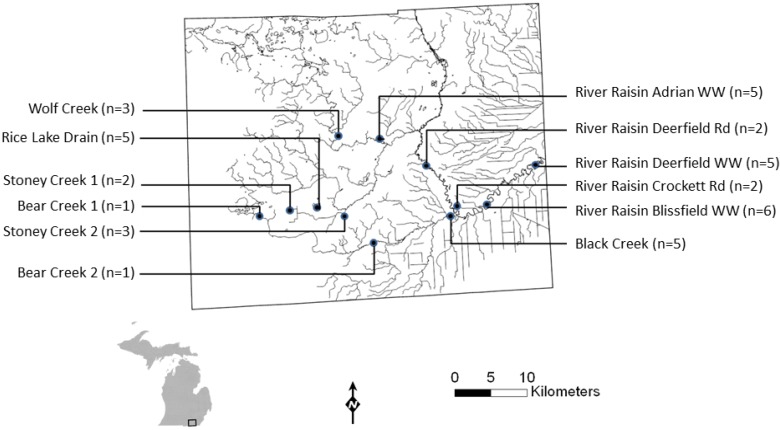
Map of the River Raisin watershed, Michigan USA where intensive *Cryptosporidium* and *Giardia* sampling was conducted from 22 October 2004 and 14 February 2005. A total of 13 sites were sampled; note that the Rice Lake Drain site included samples from two adjacent sites, a county drain and an agricultural tile drain (WW = water works).

Between 22 October 2004 and 14 February 2005, surface water samples were collected from 13 sites in the River Raisin watershed (Figure 3). The River Raisin watershed occupies an area of 2700 km^2^, with agriculture being the primary land use (~75% of watershed area). The River Raisin has been designated as an Area of Concern in the Great Lakes basin; management priorities include nonpoint source pollution control and elimination of CSOs [28]. All sampling sites except St. Joseph Creek were located within the Lenawee County portion of the River Raisin watershed. St Joseph Creek is part of the Bean Creek/Tiffin River Watershed, located in Hillsdale County, MI. Three of the sites were surface water treatment plant intakes in the River Raisin watershed. The intake at the drinking water treatment plant for the City of Adrian draws from Lake Adrian. Lake Adrian discharges into the South Branch River Raisin, upstream of the intakes at the Blissfield and Deerfield drinking water treatment plants. The remaining sampling sites included one site on St. Joseph Creek, multiple sites in three tributary creeks of the River Raisin upstream of the plant intakes, one county drain (Rice Lake Drain), and one agricultural field drainage structure in the River Raisin watershed. The Rice Lake Drain sampling location receives drainage from concentrated animal feeding operation (CAFO) properties and manure-applied fields via a field tile. Rice Lake Drain is part of the South Branch River Raisin sub-basin of the River Raisin Watershed.

Additional water samples were collected across the state between 2004 and 2014. Surface water samples were collected for several research projects [29,30], at the request of MDEQ to assess compliance with surface water regulations and at the request of MDCH to assist with an outbreak investigation [31]. Groundwater samples were collected and analyzed from private wells at the request of homeowners. These groundwater samples were often collected after individuals in the household were diagnosed with cryptosporidiosis or giardiasis. Typically, only one sample on a single date was collected for these private wells.

#### 2.2.2. Sampling Methods

All samples were collected and analyzed following USEPA Method 1623 [27]. At surface water sites, approximately 20 L of water were collected for *Cryptosporidium* and *Giardia* analysis. For private wells, larger volumes (up to 100 L) were collected. All samples were transported on ice and then stored at 4 °C until processed. Samples with turbidity levels less than 35 NTU were filtered through Envirocheck^TM^ HV filters (Pall Corp, New York, NY, USA) and then eluted according to manufacturer’s instructions. To avoid blocking the filters, high turbidity (≥35 NTU) samples were centrifuged at 1000–1100 G for 15 min instead of filtered to obtain a water concentrate from a water sample of relevant size.

Eluted volumes from filters were centrifuged to concentrate the sample as described in USEPA Method 1623 [27]. *Giardia* cysts and *Cryptosporidium* oocysts were separated from water sample concentrates by immunomagnetic separation (IMS), stained, and examined by microscopy as described in EPA Method 1623 [27].

Samples from the River Raisin watershed that tested positive for *Cryptosporidium* via microscopy were tested for infectivity using an *in-vitro* cell culture method as described by Slifko *et al*. [32]. A most probable number was computed for infectious samples using the Environmental Protection Agency’s ICR-MPNV program (version 1).

#### 2.2.3. Genotyping

For the River Raisin samples collected during 2004–2005, samples positive for *Cryptosporidium* by microscopy and PCR detection were genotyped. IMS concentrates were centrifuged (10,000 *g* for 3 min), and resuspended in 50 µL of molecular grade water. To extract DNA, water concentrates were mixed with Chelex resin-Tris-EDTA buffer (1:1 ratio; vol/vol) and subjected to eight freeze-thaw cycles (liquid nitrogen/boiling water). The sample was centrifuged and the supernatant removed for genotype analysis. Samples of molecular grade water were included randomly as negative controls during the DNA extraction procedures. PCR amplification reaction mixtures contained 1× PCR buffer (10× PCR buffer with 15 mM MgCl_2_, Qiagen, Hilden, Germany), 3 mM MgCl_2_, 60 µM (each) deoxynucleoside triphosphate, 100 nM (each) primer (500 nM in 100 µL reactions), 2.5 U of Hot Start Taq polymerase (Qiagen), and 5 and 50 µL of DNA template in total 50- and 100-µL reaction mixtures, respectively. Positive and negative PCR controls were run in parallel with each set of samples. PCR-positive controls for the initial amplification reaction consisted of *C. parvum* template DNA. PCR negative controls contained molecular-grade water. Primary and nested PCR was performed. Primary PCR was performed with primers 5'-TTCTAGAGCTAATACATGCG-3' [33] and 5'-CCCATTTCCTTCGAAA CAGGA-3' [34]. Thirty five PCR cycles (94 °C for 45 s, 55 °C for 60 s, and 72 °C for 90 s) were carried out in a thermal cycler with an initial hot start at 95 °C for 15 min and a final extension at 72 °C for 7 mins. Nested PCR was carried out under the same conditions, using 5 µL of the primary PCR product as reaction template, and nested primers 5'-GGAAGGGTTGTATTTATTAGATAAAG-3' and 5'-AAGGAGTAAGGAACAACCTCCA-3' [33]. Cycling conditions were identical to those used for the primary PCR. PCR products were analyzed on 1.5% agarose gels containing 4 µL/100 mL of Gel Star nucleic acid stain (Cambrex, Rockland, ME, USA) in 1× Tris-borate-EDTA buffer. Resulting bands were visualized by UV transillumination.

Restriction fragment analysis was performed as described by Xiao *et al.* [33]. Digested products were fractioned on a 2.0% agarose gel containing 4 µL/100 mL of Gel Star nucleic acid stain (Cambrex) and were visualized by UV transillumination. The patterns of DNA bands were used to differentiate the species and genotypes of *Cryptosporidium* parasites [33].

Nested PCR products were cloned for sequencing, using the TOPO TA cloning kit (Invitrogen, Carlsbad, CA, USA) and plasmid DNA was isolated and purified using the Wizard Plus SV Minipreps DNA purification kit (Promega, Madison, WI, USA) prior to submission for sequencing. Automated sequencing was performed on the ABI PRISM 3100 Genetic Analyzer (Applied Biosystems, Foster City, CA, USA) of the Genomic Technology Support Facility at Michigan State University. Sequencing was performed on two clones from each sample in both directions, and the resulting consensus sequences were compared (BLAST; www.NCBI.NIH.gov) with sequences available in the GenBank database to identify possible matches with the sample sequences.

#### 2.2.4. Statistical Analyses

We used the Mann-Whitney Rank Sum Test to analyze differences between the percent of positive samples for urban and rural sites and the percent of positive samples for surface and groundwater sites. We also compared differences between the percent of positive samples for urban and rural sites for just surface water samples. We chose this non-parametric analysis because the data did not meet the assumption of normality and data transformations failed to achieve normality.

### 2.3. Relationship between Disease and Land Use

To investigate patterns between land cover type and the incidence of giardiasis and cryptosporidiosis, we constructed a database in ArcGIS (ArcGIS 9.2, ESRI). The database was populated with publicly available information from the Michigan Geographic Data Library including census tracts from the counties of interest (Hillsdale, Lenawee, Ottawa, and Kent) and the 2001 land cover raster image of Michigan’s Lower Peninsula (30m × 30m cell size). The database also included information obtained from the United States Census Bureau on the five digit ZIP code tabulation areas (ZCTAs) for the year 2000 [16]. Information on giardiasis and cryptosporidiosis cases was obtained from the Michigan Department of Community Health (MDCH). Case information over the time span of January 2000 to December 2008 was summed by ZIP code and included in the database. Data on additional attributes for each ZIP code in the study area were obtained from the 2000 United States Census. These attributes were: (1) population, (2) percentage of individuals below poverty level, (3) median household income, (4) median age, (5) mean travel time to work, and (6) percentage of Caucasian/white individuals. The end product was a database with records of ZIP codes, percentage of “rural” area, percentage of “urban” area, percentage of “other” area, numbers of cases of giardiasis, and numbers of cases of cryptosporidiosis during the period from January 2000 to December 2008. ZIP codes that were ≥50% rural by area were designated as rural ZIP codes. ZIP codes that were ≥50% urban by area were designated as urban ZIP codes. For further information on database creation, see the Supplemental Materials.

Using the database, ZIP codes that overlapped into counties other than Kent, Ottawa, Lenawee, and Hillsdale counties were identified and excluded from statistical analysis of disease occurrence and land cover type. There were 102 ZIP codes identified in the Kent, Ottawa, Lenawee, and Hillsdale counties and 54 of these ZIP codes were excluded from the statistical analysis. Two of the excluded ZIP codes had neither >50% urban area or >50% rural area, five ZIP codes were generic 3 digit ZIP codes which had no population associated with them (*i.e*., areas located alongside interstate highways), two ZIP codes had no area associated with them in the ArcGIS database, and 45 of the ZIP codes extended beyond the borders of Hillsdale, Lenawee, Kent, and Ottawa county. Thus, 48 ZIP codes of the 102 ZIP codes in Kent, Ottawa, Lenawee, and Hillsdale counties were analyzed.

Information on study area attributes for each of the 48 ZIP codes was obtained from either the constructed ArcGIS database or the 2000 United States Census [16] for statistical comparison. These attributes were: (1) population, (2) area, (3) population density, (4) percentage of individuals below poverty level, (5) median household income, (6) median age, (7) mean travel time to work, and (8) percentage of Caucasian/white individuals. We compared these attributes between the urban and rural ZIP codes and examined for statistically significant differences. Datasets for statistical analysis were not normally distributed. The “Median household income” and “Percentage of individuals below poverty level” attributes could be transformed to produce normally distributed datasets using a log transformation. For these attributes, t-tests were used to examine the datasets for statistically significant differences. No transformation was found that produced normally distributed datasets for the other demographic attributes; therefore, the non-parametric Mann-Whitney Rank Sum Test was used to analyze differences between urban and rural zip codes.

Statistical analysis of urban cryptosporidiosis, rural cryptosporidiosis, urban giardiasis, and rural giardiasis was performed using ZIP codes that had been classified as either urban or rural. We first analyzed the case data (January 2000 to December 2008) for each disease, then performed the analysis on data normalized by three different methods: (1) case data for each disease normalized by the area of the ZIP code, (2) case data for each disease normalized by the population of the ZIP code, and finally (3) case data for each disease normalized by the population density of the ZIP code. We used non-parametric tests because the data were not normally distributed and no transformation was found that produced normally-distributed datasets. For each analysis, a Kruskal-Wallis One Way Analysis of Variance (ANOVA) on Ranks was performed (SigmaPlot 11, Systat Software, Inc., San Jose, CA, USA), followed by a Dunn's test to evaluate multiple pairwise differences. We used Spearman correlation on ranks to analyze relationships between the percentage of urban area and percentage of rural area of ZIP codes in the study area and seven other factors. These factors were population, population density, percentage of Caucasian/white individuals, percentage of individuals below poverty level, median household income, median age, and mean travel time to work. We considered correlations to be strong if r > 0.9 and moderately strong if r > 0.75.

## 3. Results and Discussion

### 3.1. Prevalence in the Rural and Urban Water Environments

#### 3.1.1. Occurrence of *Cryptosporidium* and *Giardia* in Rural *versus* Urban Areas

**Figure 4:**
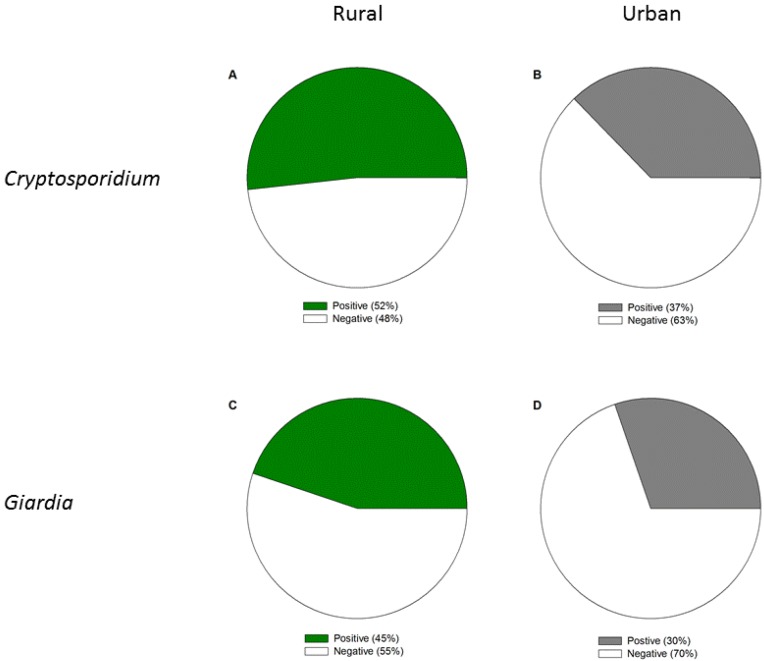
Percent of positive samples for (**A**) *Cryptosporidium* at rural sites (n = 29), (**B**) *Cryptosporidium* at urban sites (n = 51), (**C**) *Giardia* at rural sites (n = 29), and (**D**) *Giardia* at urban sites (n = 33). Samples were collected across Michigan from 2004–2014 from both surface and groundwater sites.

We detected a significant difference in the percent of samples testing positive for *Cryptosporidium* between urban and rural sites (Mann-Whitney U = 916.0, *p* = 0.05). Of the urban sites, 37% tested positive for *Cryptosporidium* whereas 52% of the rural sites tested positive (Figure 4). Surface and groundwater sites were also significantly different (U = 256.5, *p* < 0.001), with 56% surface water sites and none of groundwater sites testing positive for *Cryptosporidium*. We did not detect any differences in the concentrations of *Cryptosporidium* in positive samples between urban and rural sites or between surface and groundwater sites. We also did not detect a significant difference in the percent of samples testing positive for *Giardia* between urban and rural sites (Figure 4); however, we did detect a significant difference between surface water and groundwater sites (U = 209.0, *p* = 0.001). Of the surface water sites, 50% tested positive for *Giardia* whereas only one of the groundwater sites tested positive. The single positive groundwater sample was from a drinking water plant in an urban county using groundwater under the influence of surface water. When we excluded groundwater samples, we did not detect significant differences between surface water samples from urban and rural sites for either *Cryptosporidium* or *Giardia*. However, more samples are available from urban areas compared to rural areas.

During the 2004–2005 monitoring of rural sites in the River Raisin watershed, *Cryptosporidium* was detected in 11 of 13 surface water sites. Monitoring was undertaken because of high *E. coli* levels found in these waterways and there was interest in the occurrence of zoonotic pathogens. The site with the highest detected level of *Cryptosporidium* was the agricultural tile drain that discharges into Rice Lake Drain. *Giardia* was detected at 8 of the surface water sites. The only site to test positive for infective oocysts was the agricultural tile drain discharge into Rice Lake Drain (Table 2).

**Table 2:** Concentration and infectivity of *Cryptosporidium* and concentration of *Giardia* for the River Raisin watershed sampling sites collected 2004–2005.

Site Location	Sample Collection Dates	*Cryptosporidium* Concentration (Oocyst/100 L)	*Giardia* Concentration (Oocyst/100 L)	*Cryptosporidium* Infectivity (MPN/mL) (95% Confidence Interval)
Rice Lake Drain	10/22/2004	2600	<29	ND
	11/4/2004	582.2	<34.2	ND
Agricultural Drain Tile into Rice Lake Drain	11/9/2004	30.3	<15.2	ND
	12/6/2004	59,900	76.3	2.301 [0.1789 : 6.238]
	12/7/2004	1250	50	ND
Black Creek at Crockett Rd.	11/9/2004	<10.6	10.6	--
	11/17/2004	<9.7	<9.7	--
	12/6/2004	<9.26	<9.26	--
	12/7/2004	38	38	ND
	12/14/2004	<28.45	<28.45	--
Stoney Creek 1 (at Senecca Rd.)	11/17/2004	64.6	21.5	ND
	12/7/2004	1350	<150	ND
Stoney Creek 2 (at Gorman Rd.)	12/7/2004	450	<50	ND
	12/14/2004	<24.1	<24.1	--
St. Joseph Creek at Beecher Rd.	10/22/2004	<13.4	<13.4	--
	11/4/2004	<21.1	<21.1	--
	11/17/2004	<17.2	<17.2	--
	12/14/2004	21.8	21.8	ND
Main Branch River Raisin at Deerfield Rd	12/6/2004	<44	<44	--
	1/5/2005	60.9	40.6	ND
Main Branch River Raisin at Crockett Rd	12/6/2004	<9.050	<9.050	--
	1/5/2005	18.0	<18.0	ND
Adrian Water Works Raw Influent	1/17/2005	<15.4	<15.4	--
	1/24/2005	28.4	14.2	ND
	1/31/2005	13.5	<13.5	ND
	2/7/2005	75.8	<25.2	ND
	2/14/2005	<13.1	<13.1	--
Blissfield Water Works Raw Influent	12/14/2004	<24.1	<24.1	ND
	1/17/2005	50.6	<12.6	ND
	1/24/2005	<12.7	<12.7	--
	1/31/2005	<19.4	19.4	--
	2/7/2005	<11.6	<11.6	--
	2/14/2005	64.3	<16.1	ND
Deerfield Water WorksRaw Influent	1/18/2005	15.7	<15.7	ND
	1/24/2005	<19.0	<19.0	--
	1/31/2005	<14.7	<14.7	--
	2/7/2005	39.6	<13.2	ND
	2/14/2005	64.3	<16.1	ND
Bear Creek 1 (at Medina Rd.)	11/9/2004	<8.8	8.8	*--*
Wolf Creek at Forrister Rd.	10/22/2004	<20	<20	--
	11/4/20041	<13.1	<13.1	--
	1/17/2004	<10.3	<10.3	--
Bear Creek 2 (at Morse Rd.)	11/9/2004	<18.2	<18.2	*--*

Notes: ND—sample assayed for infectivity, no infectivity detected; “<”—sample below detection limit of assay.

#### 3.1.2. Genotyping of *Cryptosporidium* in Rural Water Samples

**Figure 5:**
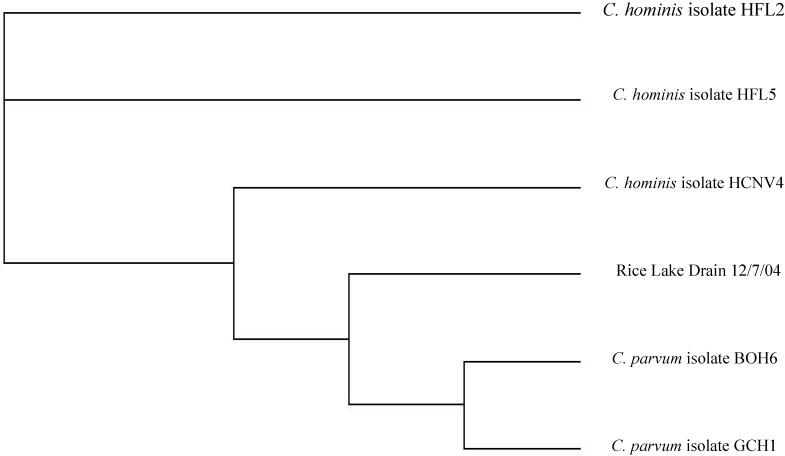
Dendrogram showing the relationship between the Rice Lake Drain 12/7/04 DNA sequence and *Cryptosporidium* sequences of known host origin and genotype. Note: The further to the right the branch is, the closer the relationship i.e. Rice Lake Drain 12/7/04 is more closely related to the two *C. parvum* sequences than to the *C. hominis* sequences.

Four samples from the River Raisin watershed were identified as *C. hominis*. This species is found only in humans, indicating a human source of fecal pollution. Four samples matched *C. parvum*, which is a species found in animals (including cattle) and humans. One sample did not match any known sequence and is likely from a wildlife source. The sample collected from the agricultural tile discharge into Rice Lake drain was identified as *C. parvum* via the RFLP pattern, and another *Cryptosporidium* sequence from the tile sample had the closest relationship to sequences of *C. parvum* genotype 2 (Figure 5). *C. parvum* genotype 2 infects humans and some other mammals, including ruminants [35,36]. The presence of *C.* *parvum* indicates a human, cattle, or mixed human and cattle source of fecal contamination in these waters. RFLP analysis showed that two samples from a small community’s drinking water intake were matched closest to *C. andersoni* and *C. parvum*, thus drinking water sources in these rural areas contain multiple *Cryptosporidium* species indicating an input of cattle fecal pollution and a potential input of human fecal pollution.

The genotyping of *Cryptosporidium* found in Michigan waterways suggests that agricultural practices are contributing to contamination of these waterways. *C. andersoni* is a species found in cattle [37] and has been previously found in both cattle and surface water from farms in the lower peninsula of Michigan [38]. *C. andersoni* infects both juvenile and adult cattle and has a long oocyst shedding duration of months to years, compared to the short shedding duration (~1–2 weeks) of *C. parvum* [37,38]. While the presence of *C. andersoni* in surface waters presents a low risk to public health, it does indicate a probable cattle source of fecal contamination in these waters and may pose a risk to farms if these surface waters are used as an animal drinking water source.

To answer our first research question, we found a higher number of positive samples for *Cryptosporidium* and *Giardia* in rural *versus* urban environments; however, we are limited by the number of samples available to draw conclusions regarding overall prevalence in urban *versus* rural environments. For households that used private wells, no parasite contamination was found in either rural (n = 4) or urban (n = 11) wells. One of the three urban centers that utilized groundwater tested positive for *Giardia* and none tested positive for *Cryptosporidium*. The highest concentrations of parasites were found in rural drains. The presence of infective *C. hominis* and *C. parvum* in tile drain discharge to the Rice Lake Drain (Table 2) indicates both agricultural and human waste may be sources of protozoa in the environment and in surface water that potentially impact drinking water supplies.

### 3.2. Relationship between Disease and Land Use

Significant differences between urban and rural ZIP codes were found in all attributes examined (*i.e*., population, area, population density, percentage of individuals below poverty level, median household income, median age, mean travel time to work, and percentage of Caucasian/white individuals). When compared to urban zip codes, the rural zip codes had a higher percentage of Caucasian/white individuals in the population, the population age was older, the mean travel time to work was longer, and the median household income was higher. Additionally, in the rural zip codes, a lower percentage of individuals were living below the poverty level than in the urban zip codes. Three of these attributes (population, area, and population density) were used to normalize the cases of disease. The attributes of population and area both had higher values in rural areas than in urban areas. In contrast, the population density was higher in urban areas than in rural areas. The three normalized datasets and the non-normalized dataset were used to test the null hypothesis that disease levels in urban and rural areas are the same.

A comparison of the total disease cases, which represents disease incidence or the number of cases in a given amount of time, by urban or rural designation over the eight year study period is shown in Table 3. In this dataset, the total number of both giardiasis and cryptosporidiosis cases were greatest in the rural areas, whereas the median values of giardiasis and cryptosporidiosis case incidence were greatest in the urban ZIP codes (data not shown). Once the incidence datasets were normalized (by population, area, or population density), the patterns of disease occurrence between the total number of cases and the median number of cases per ZIP code categories were in agreement with a more significant disease burden in the rural areas.

**Table 3:** Comparison of disease cases * from January 2000 to December 2008 by urban or rural ZIP code designation.

Variable	Cryptosporidiosis Total Cases		Giardiasis Total Cases	
	Urban	Rural	Urban	Rural
Number of Disease Cases	39 **	113 **	405 **	467 **
Cases/Population	1.3 × 10^−4^	2.4 × 10^−4^	1.3 × 10^−3^	9.8 × 10^−4^
Cases/Area	0.19 **	0.03 **	1.9 **	0.10 **
Cases/Population Density	0.03	1.1	0.28	4.5

Notes: * The dataset of disease cases represents disease incidence, which is the number of cases in a given amount of time; ** Statistical differences based on Kruskal-Wallis One Way ANOVA on Ranks; *p* < 0.001.

Statistically significant differences were present between disease occurrence in ZIP codes designated as urban and those designated as rural (Table 3, *p* < 0.001). Therefore, the null hypothesis was rejected in favor of the research hypothesis that there were significant differences between incidence of disease in urban and rural areas. This difference between rural and urban ZIP codes was maintained even when disease occurrence was normalized by different attributes present in the study area (*i.e*., population size, area, or population density of the ZIP codes). When the disease cases were divided by population size, the number of cryptosporidiosis disease cases was greatest in the rural ZIP codes. Conversely, for giardiasis, the greatest numbers of disease cases were in the urban ZIP codes although the difference was not statistically significant. When the disease cases were divided by area, the numbers of disease cases were significantly greater in the urban ZIP codes for both cryptosporidiosis and giardiasis. When the disease cases were divided by population density, the number of disease cases was greatest in the rural ZIP codes for both cryptosporidiosis and giardiasis although this difference was not statistically significant.

Three strong correlations were found between disease cases and land use and population characteristics at the ZIP code scale (Table 4). Strong positive correlations existed between giardiasis and population (r = 0.92, *p* < 0.001) and between the percentage of urban area and population density (r = 0.98, *p* < 0.001). A strong inverse relationship existed between the percentage of rural area and population density (r = −0.98, *p* < 0.001). Five moderately strong positive correlations and four moderately strong inverse correlations were found in this assessment (Table 4). The moderately strong positive correlations consisted of giardiasis and population density, giardiasis and percentage urban area, cryptosporidiosis and population, percentage urban area and population, and percentage rural area and percentage of Caucasian/white individuals. The moderately strong inverse correlations consisted of giardiasis and percentage rural area, the percentage of urban area and percentage of Caucasian/white individuals, the percentage of rural area and population, and giardiasis and mean travel time to work. The demographic characteristics (*i.e*., percentage of Caucasian/white individuals, median age and mean travel time to work) are significantly correlated to percent urban and rural area. No significant correlations with either the median household income or the percentage of individuals below poverty level were found (*p* > 0.15). The relationship with population produced the highest correlation coefficients for cases of either giardiasis or cryptosporidiosis, although the correlation with giardiasis was strong and the correlation with cryptosporidiosis was only moderate. For both giardiasis and cryptosporidiosis, the next strongest relationship was with population density.

**Table 4:** Statistically significant correlations (*p* < 0.001) between disease cases and land use and population characteristics. Correlations (r values) greater than or equal to 0.75 are shown in bold.

Factor	Total Giardiasis Cases	Total Cryptosporidiosis Cases	% Urban Area	% Rural Area
% Urban Area	**0.79**	0.60	-	-
% Rural Area	**−0.79**	−0.59	-	-
Population	**0.92**	**0.79**	**0.75**	**−0.75**
Population Density	**0.80**	0.61	**0.98**	**−0.98**
% White	−0.69	−0.55	**−0.78**	**0.76**
Median Age	−0.64	−0.49	−0.55	0.56
Mean Travel Time to Work	**−0.75**	−0.66	−0.65	0.65

Livestock density and manure land application were not included in the prototype database, although these may be important factors in disease transmission. Information on livestock density is only available at the county scale. Including this information in the prototype database would require all comparisons to be done at the county scale, potentially obscuring patterns that occur at more local levels. However, several studies in the scientific literature that have examined associations between cryptosporidosis/giardiasis and livestock density, manure application, and other livestock related factors. These studies provide insight into which attributes would be desirable to include in an expanded GIS database.

Previous studies of cryptosporidiosis and giardiasis in England, Scotland, and Wales have demonstrated association with rural areas in some studies and associations with urban areas in others. During the 2001 foot and mouth disease outbreak in livestock in England and Wales, in which control measures were implemented including restriction of access to farms, limiting movement of livestock for trade and between pasturage, and culling of affected herds and flocks, there was a corresponding decrease in reported cryptosporidiosis in humans ranging from 35–63% [39] throughout England and Wales with declines of 81.8% in northwest England [40]. During the interval of foot and mouth disease control measures, the proportion of *C. parvum* cases decreased compared to case incidence in 2000, suggesting a decrease in human infection due to a decrease in exposure to pathogen reservoirs in livestock [39]. No significant reduction in giardiasis cases was observed during this interval, which may indicate a difference between *Giardia* and *Cryptosporidium* transmission routes and reservoirs of infection [39]. Studies of area based cryptospordiosis rates in England and Wales using housing density to define rural areas demonstrated higher illness rates in rural areas than urban areas, higher rates in areas with more agricultural manure application, and higher rates in areas with inadequate drinking water treatment [14,41]. A study of spatial epidemiology of sporadic cases of human cryptosporidiosis in Scotland found increased rates of *C. parvum* infection in areas with lower human density, a higher ratio of farms to humans, and a higher ratio of private water supplies to the human population, indicating an association of *C. parvum* infection with rural areas [13]. Unlike *C. parvum, C. hominis* was reported more often in the more heavily populated areas of south Scotland, associating this genotype more strongly with urban areas [13]. In a case control study of cryptosporidiosis in the United Kingdom, the urban-rural gradient was not found to be a significant variable in the full model of disease etiology when both *C. parvum* and *C. hominis* cases were included. However, when *C. hominis* cases were excluded, cryptosporidiosis was negatively associated with urban areas and when *C. parvum* cases were excluded, cryptosporidiosis was positively associated with urban areas, indicating genotype specific transmission associated with the geographical classifications [41]. The differences in the association of cryptosporidiosis with urban or rural areas in the scientific literature may therefore be partially due to the *Cryptosporidium* genotype causing the infections.

In a spatial investigation of giardiasis in Canada that explored associations with livestock density and land application of manure with disease patterns, low correlation coefficients between giardiasis rates and cattle density (r = 0.11) and between giardiasis rates and land application (r = 0.09) were observed when all geographic regions in the study area were included [15]. However, these correlation coefficients were higher in certain regions of the study area when disease rates were examined at smaller spatial scales [15].

These results suggest that livestock density and land application of manure can contribute to transmission of *Giardia*, but that other factors may be more important. In the current study, the major caveat to the conclusion that significant differences in the patterns of giardiasis and cryptosporidiosis exist between urban and rural ZIP codes is the small number of ZIP codes that are designated as urban in the study area. Because this method of examining health data appears promising, expanding the study area to larger geographic regions of the United States (*i.e*., the Midwest) in future work is recommended. Expanding the geographic region would also allow for agricultural census data to be incorporated into the study design by allowing analysis at the county level, which is the minimum scale of the agricultural census data. Previous studies from the scientific literature suggest that livestock density, animal transport frequency, the number of farm workers/visitors, manure application rate, ratio of farms to humans, and the *Cryptosporidium* genotype would be informative attributes in an expanded GIS database. Utilizing GIS as a tool to integrate factors from the rural environment with factors from the urbanized environment has the potential to be extremely useful to public health agencies in targeting funds to reduce disease transmission in communities.

To summarize our results regarding our second research question, we found significant differences in the number of reported disease cases from 2000–2008 between urban and rural areas. Rural areas had a higher incidence of disease compared to urban areas. Cryptosporidiosis and giardiasis were both significantly and positively correlated urban area, population size and population density.

### 3.3. Lessons Learned: Implications for Management

Managing human health risks from *Cryptosporidium* and *Giardia* has been largely reactionary. In Michigan, the current strategy for managing these pathogens has been driven by federal regulations for drinking water systems and responses to outbreaks. Federal regulation in the US was spurred by the *Cryptosporidium* outbreaks in Milwaukee, Wisconsin and Carrolton, Georgia [42]. One result was creation of the LT2 rule and monitoring program. A similar pattern was described in the UK by Austin *et al.* [43], where evolving management practices for *Cryptosporidium* were triggered by high profile outbreaks which in turn triggered regulation. The additional regulation spurred advances in technological methods for monitoring *Cryptosporidium* that ultimately provided information necessary for improved risk management [43]. The monitoring results provided managers with information regarding how frequent raw water samples tested positive, where source waters tested positive, and uncovered uncertainties related to risk such as if positive samples were infectious to humans. These results informed risk assessments and development of water safety plans [43]. The authors conclude that continuous assessment and improvement in decision-making is required to prevent the negative health and social effects of disease [43]. Central to this improvement in decision-making for *Cryptosporidium* and *Giardia* is access to data and information regarding where the threats are and how best to manage them.

Our limited analysis reveals that surface waters in rural areas have a higher occurrence of positive samples and a greater number of disease cases. However, transmission through exposure to contaminated surface water, animals, animal wastes/manure and septage or septic tank discharges was not revealed. Oocysts and cysts have limited movement in soils and groundwater aquifers; however, our understanding of risks due to groundwater contamination is limited by the paucity of information collected from wells. Exposure to contaminated surface water in these rural environments is a known risk, as demonstrated by a recent outbreak of cryptosporidiosis in firefighters who were exposed to pond water that tested positive for *Cryptosporidium* [31]. Greater treatment of manure and septage may be warranted to limit contamination of waters in rural environments used for livestock and irrigation. Heat and composting are effective against oocysts and cysts compared to high lime pH treatment [44,45,46].

A greater understanding of the quality of groundwater in rural environments is needed. In Michigan, there are no requirements for water quality testing of private wells beyond those required during well construction. The MDEQ, MDCH, and local health departments rely on public education programs for private well owners and recommend testing well water annually for coliform bacteria or if color, odor or taste abruptly changes. However, it is unclear if or how often private well owners test their wells. Community water systems in rural areas are regulated by the Safe Drinking Water Act, but they are often small (serve less than 3300 people) or very small systems (serve less than 500 people) with limited financial and human resources. These conditions are exacerbated by a general lack of resources in rural areas. Rural counties in Michigan have higher percentages of unemployment and poverty compared to urban counties [18]. Comprehensive monitoring using expensive tests, such as those required for *Cryptosporidium* and *Giardia*, is not a feasible option for many private well owners and rural community water systems that lack financial and technical resources.

Our analysis also found key gaps in available water quality data. In order to fill data gaps and provide technical assistance to rural areas, a comprehensive monitoring strategy for *Cryptosporidium* and *Giardia* is needed. Because such an effort is likely to be resource-intensive, we recommend the use of GIS to identify sampling sites in areas that currently lack data or in areas experiencing high incidence of disease. Monitoring efforts should prioritize groundwater and rural areas given the higher number of disease cases and higher number of positive samples in rural areas. We also encourage the use and development of new monitoring technologies that reduce monitoring costs and provide critical information, such as genotype and infectivity in addition to presence/absence.

**Figure 6:**
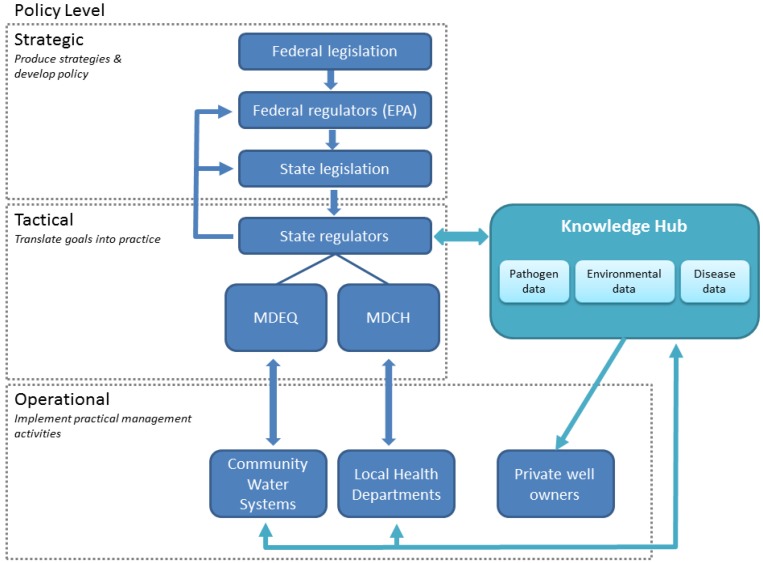
Schematic diagram of strategy to create a knowledge hub that facilitates pathways for data sharing among multiple levels of managers to improve decision-making for protecting rural water supplies. Knowledge pathways and policy levels based on Austin *et al.* [43]. MDEQ = Michigan Department of Environmental Quality; MDCH = Michigan Department of Community Health.

In addition to monitoring in order to fill data gaps, we recommend creating an easily-accessible, online knowledge hub for information sharing to support management and communication at multiple levels (Figure 6). Ideally, the knowledge hub would be a spatial database that includes environmental data, water monitoring data, and disease reporting and epidemiological data for the state. As with many jurisdictions, databases for each of these types of data currently exist; however, they are not linked. Exploring relationships among environmental, water quality and disease data can provide insight for risk management. For example, a Wisconsin study that used epidemiological data, landscape information based on public records, and water quality data demonstrated that septic system holding tank density in a rural area is a risk factor for diarrheal disease in children [47]. Our own case study also demonstrated how GIS-based analysis can help identify relationships between disease and landscape variables to help target management. A knowledge hub would potentially improve data sharing and provide information for decision-making, particularly at lower levels of government and in rural areas without the capacity to create such a resource for their own jurisdictions. Michigan already has a web-based system, Michigan BeachGuard [48], which is used for recreational water quality data that could serve as a model. The MDEQ maintains and manages the online database, which they use to assess beach water quality and attainment of water quality standards. Data are uploaded by local health departments through a secure site on BeachGuard. The public can also log onto the system to gain information on beach closures and access historical data. The knowledge hub could operate in a similar fashion, in which the state, or those in the tactical policy level, manages the database and those in the operational policy level input and/or export information (Figure 6). The tactical policy level would also have ready access to information in the knowledge hub to report to and inform groups in the strategic level. Thus, the system could facilitate information sharing while supporting management duties at multiple levels. Such a resource could also assist in filling the current management gap regarding private well owners by providing information on disease and pathogen occurrence. For example, a private well owner might log into the system to access maps showing areas of with positive samples for *Cryptosporidium* and *Giardia* or high disease rates and be able to assess if their own well is located in a potential hot spot. This may assist them in making more informed decisions about testing their wells. Finally, a geospatial database would enable managers to examine regional patterns and potentially move to regional or watershed-based management.

To summarize, our analysis highlights the need to identify potential risks and create mechanisms to improve information gathering and sharing to protect rural water supplies. Because we found that surface waters in rural areas have a higher prevalence of parasite contamination and greater disease incidence, policies that require or promote greater treatment of manure and septage may be warranted to protect human and animal health. We also identified several major data gaps, including lack of information about the quality of groundwater in rural environments and lack of comprehensive spatial and temporal data. In order to fill data gaps and provide technical assistance to rural areas, a comprehensive monitoring strategy that incorporates and links environmental data, disease data, and water quality data in a spatial database is needed. We recommend creating an easily-accessible, online knowledge hub for information sharing to support management and communication at multiple levels (Figure 6).

## 4. Conclusions

We compiled all known sources of *Cryptosporidium* and *Giardia* data available for the state of Michigan to explore differences between urban and rural areas. *Cryptosporidium* and *Giardia* were more commonly found in samples from rural areas and in surface water. Only one groundwater sample tested positive for *Giardia*; however, the number of groundwater samples was limited. Rural areas had a higher incidence of cryptosporidiosis and giardiasis compared to urban areas. There is a need for further monitoring to fill spatial and temporal data gaps regarding the occurrence of *Cryptosporidium* and *Giardia*, particularly in groundwater and in rural environments. Of particular concern is elucidating the exposure pathways in rural environments in order to design more effective management strategies.

Rural environments in Michigan, and many areas around the globe, pose a particular concern for cryptosporidiosis and giardiasis because the majority of residents drink water from untreated drinking wells. Groundwater sources are typically assumed to be safe, however the total number of cases for both cryptosporidiosis and giardiasis are higher in rural *versus* urban environments. A complicating factor for management is the lack of regulation on private drinking well water quality and the lack of resources available in rural areas. We recommend a state-wide monitoring strategy and knowledge hub to share information critical for managing human health risks from *Cryptosporidium* and *Giardia*.

## Supplementary Files

Supplementary File 1
