# Supplementary material for: Cryptosporidium and Giardia in Surface Water: A Case Study from Michigan, USA to Inform Management of Rural Water Systems
Source: Int J Environ Res Public Health. 2014 Oct 13;11(10):10480–503. doi: 10.3390/ijerph111010480 (PMC4210991; doi:10.3390/ijerph111010480)
Supplement: Supplementary File 1 [file ijerph-11-10480-s001.pdf]

Supplementary Information

***Cryptosporidium* and *Giardia* in Surface Water: A Case Study from Michigan, USA to Inform Management of Rural Water Systems**

Creation of Case Information Database Using ArcGIS

A case information table with cases of cryptosporidiosis and cases of giardiasis summed by ZIP code was created based on information from the MDCH (MDCH, personal communication). In ArcGIS, the census tract information for Kent, Ottawa, Lenawee, and Hillsdale counties was combined with the 2001 land cover of Michigan's Lower Peninsula to produce an output file containing the land cover of Kent, Ottawa, Lenawee, and Hillsdale counties. Land cover was reclassified following the reclassification decision tree shown in Figure S1 and reclassification scheme shown in Table S1 to allow hypothesis testing of the differences between urban and rural land cover. One exception to the reclassification scheme shown in Table S1 is the "Parks / Golf courses" category. This is an ambiguous category as parks could range from city parks well connected to sewer infrastructure and drinking water distribution networks located in high population density areas to remote parks with limited sewerage and drinking water access. In this case, "Parks / Golf courses" was reclassified as "urban" because this category is associated with human activity rather than agricultural livestock.

The reclassified file was combined with census tract information and the Census 2000 5-Digit ZIP Code Tabulation Areas (ZCTA). The case information from the MDCH table was added to the output file based on the ZCTA field. The land cover file and the output containing the case information were used to produce a field with information on the percentage of "rural", "urban", and "other" land cover for each ZIP code in the counties of interest, this information was then added to the database. The end product was a database with records of ZIP codes, percentage of "rural" area, percentage of "urban" area, percentage of "other" area, and numbers of cases of giaridasis and numbers of cases of cryptosporidiosis during the period from January 2000 to December 2008. ZIP codes that were ≥50% rural by area were designated as rural ZIP codes. ZIP codes that were ≥50% urban by area were designated as urban ZIP codes. Information on data files used in the creation of the ArcGIS database is shown in Table S2. Figure S2 shows the data model of the analysis process.

Figure S1. Land cover reclassification decision tree

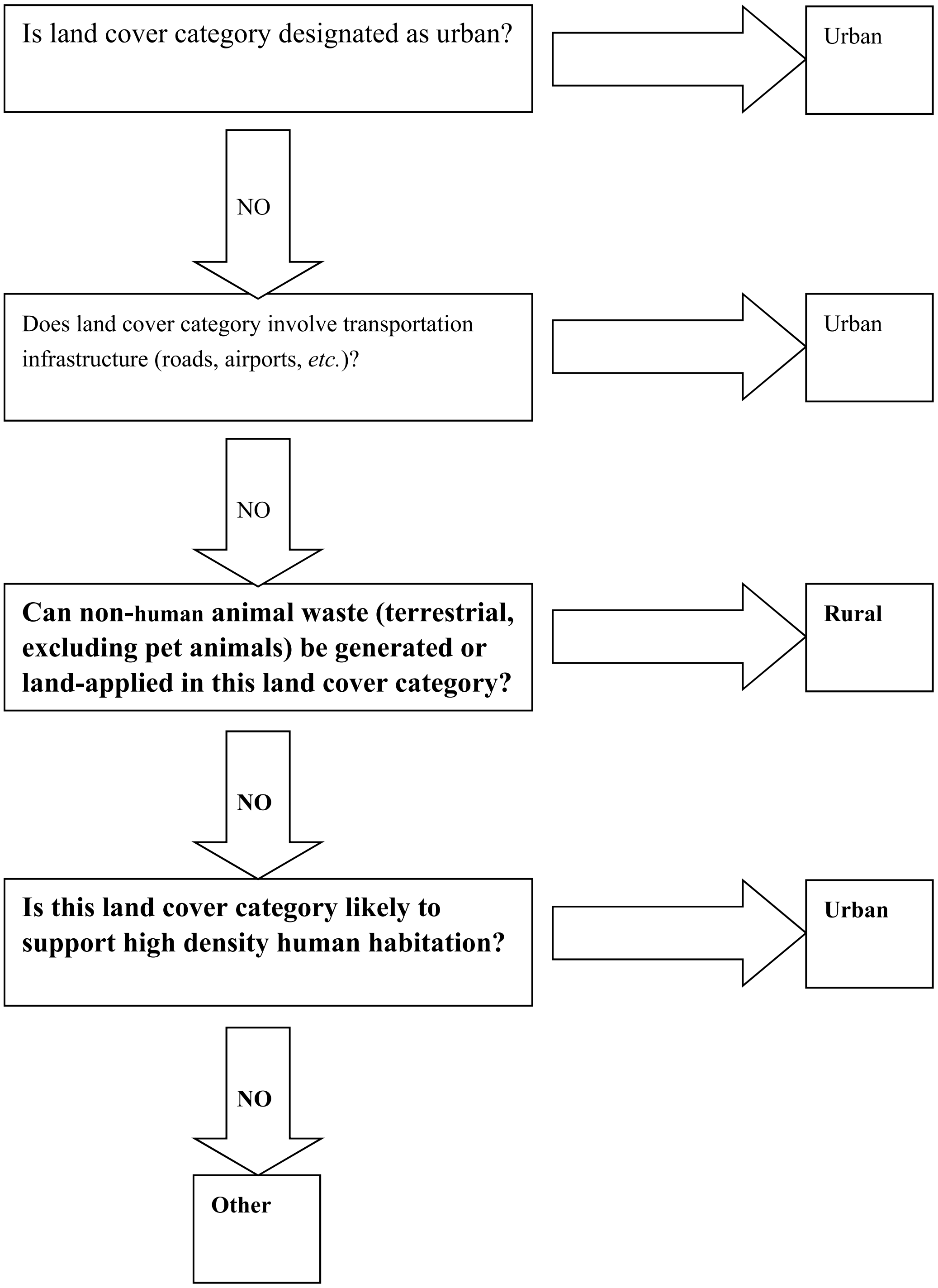

Table S1. Land cover reclassification scheme.

2001 Land cover category	Reclassified category
Non-vegetated Farmland	Rural
Forage Crops/Non-tilled Herbaceous Agriculture	Rural
Orchards/Vineyard/Nursery	Rural
Herbaceous Openland	Rural
Upland Shrub / Low density Trees	Rural
Oak Association	Rural
Aspen Association	Rural
Other Upland Deciduous	Rural
Mixed Upland Deciduous	Rural
Pines	Rural
Other Upland Conifers	Rural
Mixed Upland Conifers	Rural
Upland Mixed Forest	Rural
Lowland Deciduous Forest	Rural
Lowland Coniferous Forest	Rural
Lowland Mixed Forest	Rural
Lowland Shrub	Rural
Emergent Wetland	Rural
Mixed Non-Forest Wetland	Rural
Low Intensity Urban	Urban
High Intensity Urban	Urban
Airport	Urban
Road / Parking Lot	Urban
Parks / Golf Courses	Urban
Floating Aquatic	Other
Sand / Soil	Other
Water	Other
Exposed Rock	Other
Mud Flats	Other
Other Bare / Sparsely vegetated	Other

Table S2. ArcGIS database source files.

Data Type	Coordinate System		Age of Dataset	Reference
	Geographic	Projection		
Case information by ZIP code	N/A	N/A	01/01/2000–12/31/2008	34
2001 Land cover Lower Peninsula (Raster)	North American Datum of 1983	Michigan GeoRef	Constructed from image dates 1997–2001	36
River Raisin Watershed	North American Datum of 1983	Michigan GeoRef	1998	36
Grand River Watershed	North American Datum of 1983	Michigan GeoRef	1998	36
Census 2000 5-Digit ZIP Code Tabulation Areas (ZCTAs)	North American Datum of 1983	Michigan GeoRef	1/1/2000	51
Census 2000 Tracts Hillsdale county	North American Datum of 1983	Michigan GeoRef	5/1/2009	36
Census 2000 Tracts Ottawa county	North American Datum of 1983	Michigan GeoRef	5/1/2009	36
Census 2000 Tracts Kent county	North American Datum of 1983	Michigan GeoRef	5/1/2009	36
Census 2000 Tracts Lenawee county	North American Datum of 1983	Michigan GeoRef	5/1/2009	36

Figure S1. Ives.2 ArcGIS analysis process data model.

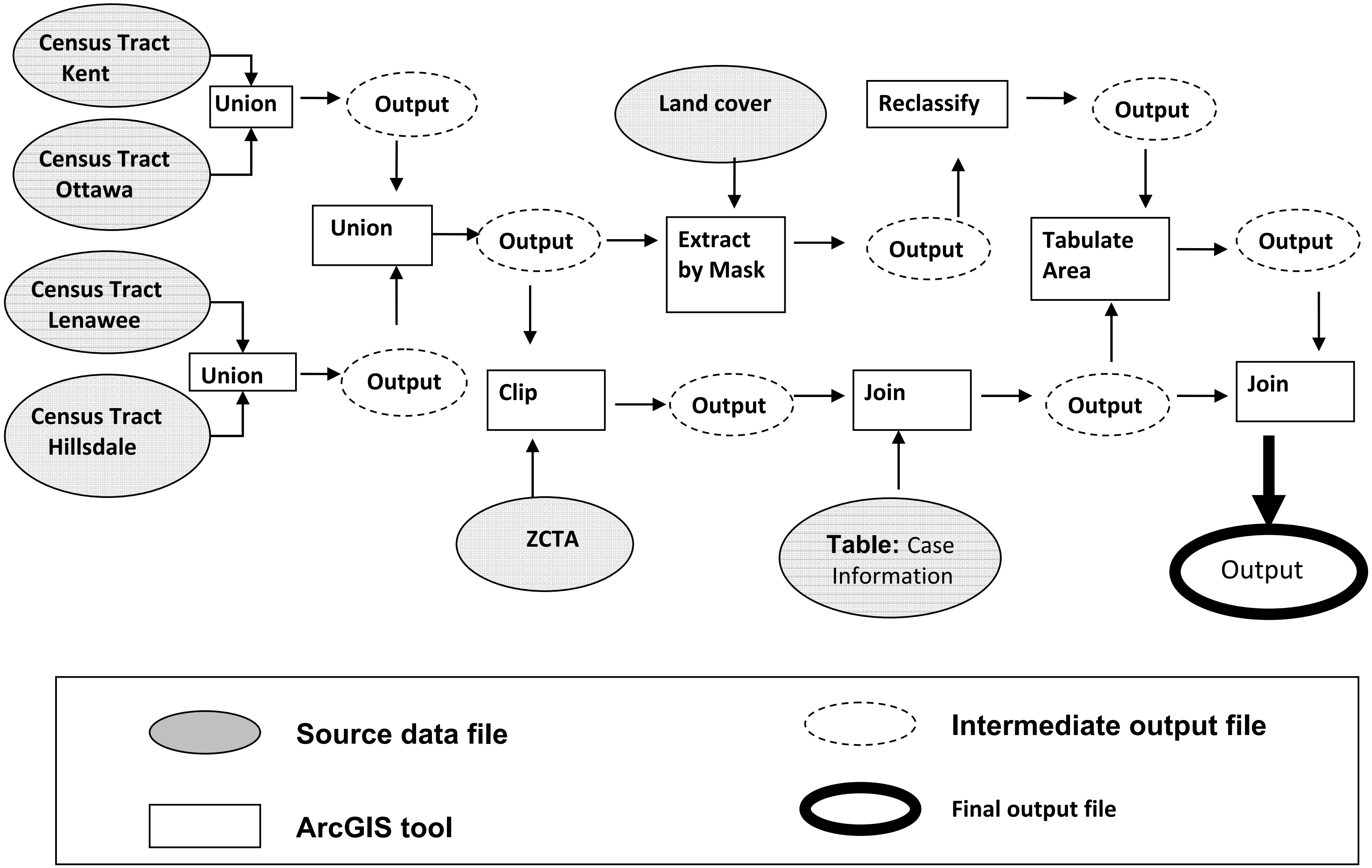

© 2014 by the authors; licensee MDPI, Basel, Switzerland. This article is an open access article distributed under the terms and conditions of the Creative Commons Attribution license (http://creativecommons.org/licenses/by/4.0/).
